# Combination of machine learning-based bulk and single-cell genomics reveals necroptosis-related molecular subtypes and immunological features in autism spectrum disorder

**DOI:** 10.3389/fimmu.2023.1139420

**Published:** 2023-04-24

**Authors:** Lichun Liu, Qingxian Fu, Huaili Ding, Hua Jiang, Zhidong Zhan, Yongxing Lai

**Affiliations:** ^1^ Department of Pharmacy, Fujian Children’s Hospital, Fuzhou, China; ^2^ Department of Pediatric Endocrinology, Fujian Children’s Hospital, Fuzhou, China; ^3^ Department of Rehabilitation Medicine, Fujian Children’s Hospital, Fuzhou, China; ^4^ Department of Pediatric Intensive Care Unit, Fujian Children’s Hospital, Fuzhou, China; ^5^ Department of Geriatric Medicine, Shengli Clinical Medical College of Fujian Medical University, Fujian Provincial Hospital, Fuzhou, China

**Keywords:** single-cell, autism spectrum disorder, necroptosis, molecular subtype, machine learning, immune infiltration

## Abstract

**Background:**

Necroptosis is a novel form of controlled cell death that contributes to the progression of various illnesses. Nonetheless, the function and significance of necroptosis in autism spectrum disorders (ASD) remain unknown and require further investigation.

**Methods:**

We utilized single-nucleus RNA sequencing (snRNA-seq) data to assess the expression patterns of necroptosis in children with autism spectrum disorder (ASD) based on 159 necroptosis-related genes. We identified differentially expressed NRGs and used an unsupervised clustering approach to divide ASD children into distinct molecular subgroups. We also evaluated immunological infiltrations and immune checkpoints using the CIBERSORT algorithm. Characteristic NRGs, identified by the LASSO, RF, and SVM-RFE algorithms, were utilized to construct a risk model. Moreover, functional enrichment, immune infiltration, and CMap analysis were further explored. Additionally, external validation was performed using RT-PCR analysis.

**Results:**

Both snRNA-seq and bulk transcriptome data demonstrated a greater necroptosis score in ASD children. Among these cell subtypes, excitatory neurons, inhibitory neurons, and endothelials displayed the highest activity of necroptosis. Children with ASD were categorized into two subtypes of necroptosis, and subtype2 exhibited higher immune activity. Four characteristic NRGs (TICAM1, CASP1, CAPN1, and CHMP4A) identified using three machine learning algorithms could predict the onset of ASD. Nomograms, calibration curves, and decision curve analysis (DCA) based on 3-NRG have been shown to have clinical benefit in children with ASD. Furthermore, necroptosis-based riskScore was found to be positively associated with immune activation. Finally, RT-PCR demonstrated differentially expressed of these four NRGs in human peripheral blood samples.

**Conclusion:**

A comprehensive identification of necroptosis may shed light on the underlying pathogenic process driving ASD onset. The classification of necroptosis subtypes and construction of a necroptosis-related risk model may yield significant insights for the individualized treatment of children with ASD.

## Introduction

Autism spectrum disorders (ASD) is a highly heterogeneous and complex neurodevelopmental disorder that manifest as deficits in social interaction, persistent impairments in behavioral or interest constrictions, stereotypy, and repetitive patterns (1). Over the past decades, the worldwide prevalence of ASD has increased significantly, recent studies have reported that ASD affects 1 in 54 children worldwide (2). Males are notably more likely to develop ASD than females (3). Recent studies have proven that genetics, inflammatory response, oxidative stress, and hypoxic insult exert crucial roles in leading to ASD progression (4–7). The specific pathological mechanisms underlying ASD remain largely unexplored. Moreover, individual differences in ASD children are the primary factor leading to the poor efficacy of drug therapy in clinical practice. Therefore, it is urgent to explore novel molecular mechanisms and therapeutic targets for the early diagnosis and treatment of ASD.

Necroptosis is a novel non-caspase-mediated regulated cell death, and is characterized by cell swelling, cytoplasmic vacuolization, and stressful rupture of cell membranes (8, 9). Under pathological conditions, necrotic cells activate the inflammatory response in surrounding cells by releasing the contents of cytoplasm, thus playing a crucial role in inflammatory diseases, including sepsis, systemic lupus erythematosus, and rheumatoid arthritis (10–13). In addition, a growing number of studies demonstrated that necroptosis can also serve as a key regulator in promoting the onset of central nervous system (CNS) diseases. For example, RIPK-mediated necroptosis may induce the microglial activation and eventually lead to retinal degeneration (14). Necroptosis-related markers, such as RIPK1, RIPK3, and MLK, exhibited a region-specific enhancement in the brain with age, suggesting their role in age-related cognitive impairment (15). Moreover, necroptosis may act as a promising target for the treatment of stroke, as the neuroprotective effects of genetic or pharmacological inhibition of necroptosis against stroke have been demonstrated *in vitro* and *in vivo* (16). However, the role and molecular mechanism of necroptosis in ASD remain unknown and need further elucidation.

In the current study, we comprehensively identified the patterns of necroptosis in ASD children. Data from single-nucleus RNA sequencing (snRNA-seq) were employed to visualize the necroptosis landscape in ASD children and among various cell types. ASD children were subsequently classified into heterogeneous subtypes based on the differentially expressed necroptosis-related genes (NRGs), and the biological functions, necroptosis levels, and immunological features between subtypes were further evaluated. Subtype-specific NRGs were identified using weighted gene co-expression network analysis (WGCNA). Three machine learning algorithms (least absolute shrinkage and selection operator (LASSO), random forest (RF), and support vector machine-recursive feature elimination (SVM-RFE)) were employed to determine the characteristic NRGs. For ASD children at distinct risk levels, a necroptosis-based scoring system was constructed to explore their biological characteristics, immune score, and predictive drugs. Finally, RT-PCR analysis was employed to further validate the expression of distinctive NRGs. The current study inventively elucidated the relationship between necroptosis expression landscapes and ASD heterogeneity, providing innovative insights for the individualized treatment of children with ASD.

## Materials

### Data acquisition and processing

Three bulk transcriptomic datasets related to ASD (GSE111176, GSE18123, GSE42133) were obtained from the Gene Expression Omnibus (GEO) online website utilizing the “GEOquery” R package (17). The GPL10558 dataset GSE111176, which included 119 ASD and 126 control samples, was selected as the test set. Another GPL10558 dataset GSE42133, which included 91 ASD and 56 control subjects, and the GEP570 dataset GSE18123, which included 66 ASD and 33 normal subjects, were selected as the validation sets. These three original datasets were pre-processed and normalized using the normalizeBetweenArrays method based on the limman R package. The threshold p-value was determined by controlling for the false discovery rate (FDR).

The original single-cell transcriptomic data (41 cortical samples from 16 controls and 15 ASD patients) were obtained from the GEO database (accession number: PRJNA434002) (18). We constructed Seurat objects for total and individual cell types in the single-nucleus RNA sequencing (snRNA-seq) gene expression matrix using the Seurat package in R with min.cells = 3 and min.feature = 200. Cells with greater than 15% mitochondrial content, less than 200 genes, and more than 7000 genes were excluded. The expression landscape was normalized using the NormalizeData function. Then, the highly variable genes were identified using the FindVariableFeatures function of the Seurat package. Principal component analysis (PCA) was conducted based on the top 2000 variable genes using the RunPCA method. Clustering was performed utilizing the FindClusters function. A uniform manifold approximation and projection (UMAP) analysis was employed to further summarize the top principal components by reducing their dimensionality. Sample integration and batch elimination were performed using the RunHarmony function of the harmony package. With the use of the FindAllMarkers function, we identified differentially expressed genes (DEGs) in distinct clusters and annotated the corresponding cell types according to known cell markers. New cluster names were updated using the RenameIdents function, and the DimPlot function was employed to depict the profile of all cell types or subtypes.

### Cell-cell communication analysis

The CellChat objects were generated using the CellChat R package(19) based on the normalized snRNA-seq data from the control and ASD groups, and the CellchatDB.human ligand-receptor interaction data was utilized to set the secreted signaling pathways as the preference database. Using the default parameters, an analysis of intercellular communication was performed. Then, for comparison purposes, the CellChat objects from the control and ASD groups were merged according to the mergeCellChat function. The differential interaction strength and number between two groups were visualized using the netVisual_diffInteraction function of the CellChat package. In addition, the distribution of differentially expressed signaling pathways were determined and plotted using the rankNet function.

### Enrichment analysis at the snRNA-seq level

The irGSEA package is an integrated framework for assessing the necroptosis score of a snRNA-seq matrix. The AUCell, AUCell, singscore, and ssgsea approaches were employed to calculate the necroptosis score for each cell. The distribution of necroptosis levels in each cell was visualized using the irGSEA.density.scatterplot function of the irGSEA package.

### Identification of differentially expressed NRGs

A total of 159 NRGs were obtained from the Kyoto Encyclopedia of Genes and Genomes (KEGG) Pathway databases. The differentially expressed genes (DEGs) associated with necroptosis were screened based on the following criteria: adjusted p-value<0.001. The profiles of differentially expressed NRGs were visualized using the heatmap, volcano plot, and violin chart.

### Functional enrichment analysis at the bulk transcriptome level

The GO (Gene Ontology) and KEGG analysis were performed to assess the biological functions and signaling pathways of the differentially expressed NRGs using the “clusterProfiler” R package (20). Significant enrichment functions and pathways were determined based on the adjusted p-values < 0.05.

The “GSVA” R package was applied for evaluating the differences in enriched functions and signaling pathways between distinct necroptosis subtypes (21). Briefly, two gensets (“c2.cp.kegg.v7.4.symbol” and “c5.go.bp. v7.5.1.symbols”) obtained from the Molecular Signature Database (MSigDB) database were considered as input files for the following GSVA analysis. Relative functions and pathways were identified by calculating the GSVA scores between distinct necroptosis subtypes based on the “limma” R package. Functions and pathways with a GSVA score (|t value|) greater than 2 were considered to be significantly enriched.

GSEA is a computational algorithm based on pre-defined gene sets that calculates the difference in gene distribution between two groups. The “clusterProfiler” and “GSEABase” R packages were utilized to identify signaling pathways that were significantly enriched between different groups. The”c2.cp.kegg.v7.4.symbols” file was selected as the reference gene list. A p-value less than 0.5 was considered to be statistically significant.

### Immune cell infiltration analysis

The proportions of 22 immune cell subtypes were estimated using the CIBERSORT algorithm based on R software (22). The LM22.txt file with the gene expression matrix of 22 immune cell subtypes and the gene expression profile of each sample was selected as the input file for further analysis. Subsequently, the estimated composition ratios of 22 immune cell subtypes in each sample were visualized. In addition, we also applied the “estimate” R package for calculating the overall immune score in the ASD and control groups, respectively. A p-value less than 0.05 was considered to be statistically significant.

### Unsupervised clustering of ASD patients

The unsupervised clustering analysis was performed based on the expression profiles of differentially expressed NRGs. Briefly, using the “ConsensusClusterPlus” R package (23), 129 ASD patients were divided into different clusters using a k-means algorithm with 1000 iterations. We selected the maximum number of subtypes (k = 6), and the optimal number of subtypes was comprehensively evaluated based on the cumulative distribution function (CDF) curves, consensus matrix, and consensus clustering scores of each subtype (>0.9). In addition, t-Distributed Stochastic Neighbor Embedding (tSNE) analysis was conducted to demonstrate and visualize the distributional differences between necroptosis subtypes.

### The weighted gene co-expression network analysis

We utilized the “WGCNA” R package (24) to construct co-expression modules and screen disease target candidates. The expression profiles of the top 25% of genes with the largest variance were selected as the input data. The soft threshold power was evaluated and employed to derive an adjacency matrix representing an approximate scale-free topology (R^2^ > 0.85). Linear correlations between gene pairs were calculated using the Pearson correlation coefficient. The power function was used to convert the expression matrix to a signed adjacency matrix, build a scale-free network, and transform it into a topological overlap matrix (TOM). We then conducted hierarchical clustering of highly co-expressed genes. Branches were then removed from the cluster tree using a dynamic tree pruning algorithm to generate relative modules. The first major component is the Eigengene Module (ME), which summarizes the overall levels of gene expression in each module. MEs were employed to evaluate the association between modules and clinical traits. Module importance (MS) represents the correlation between various genetic modules and disease characteristics. Genetic significance (GS) was used to exhibit correlations between related modules and module members.

### Selection of characteristic genes

Three machine learning algorithms, least absolute shrinkage and selection operator (LASSO), random forest (RF), and support vector machine-recursive feature elimination (SVM-RFE), were employed to screen characteristic NRGs. The LASSO algorithm was employed to identify the valuable predictive genes (25, 26) and their coefficients were determined by the best penalty parameter λ related to the smallest 5-fold cross validation *via* the “glmnet” R package (27). SVM-RFE was also applied for selecting the key feature genes. A linear support vector machine assigns an appropriate weight to each feature variable, recursively filters smaller and smaller feature subsets, and uses RFE to select the optimal feature subset (28, 29). The SVM-RFE algorithm was performed through 5-fold cross-validation based on the R package of “e1071”. Boruta is a RF packed feature selection algorithm for identifying all reliable variables in a classification framework. We applied significance thresholds maximum iterations = 300 and p-value ≤ 0.01 for identifying the important feature genes based on the “Boruta” R package (30). After 300 iterations are completed, NRGs that were still not sure if they were classified as significant variables, along with NRGs rejected by the algorithm would not be included in the subsequent analysis. We then fitted the important NRGs identified by the Boruta algorithm into a RF model using the “caret” R package. Parameters were set to default, and the top 10 NRGs with gene importance were identified as RF-related feature genes. The final hub NRGs were determined by intersecting the feature genes identified by the LASSO, RF, and SVM-RFE machine learning algorithms.

### External validation of diagnostic model based on final hub NRGs

Other two datasets, GSE18123 and GSE42133, were employed to verify the accuracy of the constructed LASSO model for the diagnosis of ASD based on final hub NRGs. The “pROC” R package was applied for plotting the ROC curves for each dataset, and the AUC value was calculated to validate the classification efficiency.

### Construction of a nomogram

On the basis of the “rms” R package, the final hub NRGs were determined as the input data for establishing a nomogram gene map. The efficiency and clinical significance of the nomogram were evaluated using the calibration curves and decision curve analysis (DCA), respectively.

### Construction of the necroptosis score

The coefficients of the final hub NRGs generated from the LASSO model were employed to calculate the necroptosis score as follows: necroptosis score = Σi Coefficients_i_ × Expression level of gene_i_. The cutoff value was determined based on the median value of necroptosis score. Subsequently, 129 ASD patients were classified into high- and low-risk groups.

### Connectivity map and mechanism of action analysis

The Connectivity Map database was utilized to predict the potential small-molecule compounds targeting the riskScore. Briefly, the top 100 most upregulated and downregulated genes between high- and low-risk groups were chosen as input data. The drug signature information obtained from the CMap database was selected as the preferred drug information. The eXtreme Sum (XSum) algorithm was utilized to compared the similarity of gene expression and drug signatures, and the computed CMap scores were employed to evaluate therapeutic potential in different risk cases.

### Blood sample collection and processing

This study was approved by the Ethics Committee of Fujian Children’s Hospital. Overall, a total of 10 children with ASD (3 girls and 7 boys; mean ± standard deviation age, 3.9 ± 0.83 years) and 10 healthy volunteers (4 girls and 6 boys) matched for age (3.5 ± 1.1 years) from The Fujian Children’s Hospital were enrolled into the study. All the patients/volunteers provided written informed consent to participate in this study. The plasma samples were collected from each individual and stored on ice, then immediately transferred to the laboratory.

### RT-PCR analysis

Total RNA was isolated from Trizol reagent (Invitrogen, CA, USA). Following the manufacturer’s procedure, cDNA was produced using the PrimeScriptTM RT Reagent Kit with gDNA Eraser (No. RR047A, Takara, Shiga, Japan). SYBR Green and CFX96TM Real-Time PCR Detector Devices were used for running the real-time PCR analysis (Bio-Rad, CA, USA). After normalization to β-actin, the mRNA levels of characteristic genes were determined using the 2^-ΔΔCt^ method based on the manufacturer’s procedure. The primers utilized for the RT-PCR analysis were as follows: TICAM1: forward, 5′- ATACCACCTCTCCAAATACCAAG -3′, reverse, 5′- CGTGGAGGATCACAAAGTTATAG-3’; CASP1: forward, 5′- GGGACTCTCAGC AGCTCCTC-3′, reverse, 5′-TGCAGATAATGAGAGCAAGACG-3’; CAPN1: forward, 5′-AGTTCATCAACCTGCGAGAGG -3′, reverse, 5′-TTCTCGTCAATCTC CTCTTCTGAG-3’; CHMP4A: forward, 5′- ATTCAACAGGAGCTACAAA CAGC′, reverse, 5′- GAAACTCCAGGGTGGATAATGT-3′; β-actin: forward, 5′- GTCCACC GCAAATGCTTCTA′, reverse, 5′- TGCTGTCACCTTCACCGTTC -3′.

### Statistical analysis

All statistical analysis was conducted using R software (version 4.1.0). Wilcoxon sum-rank testing or student’s t-testing was employed to compare the difference between two groups. The correlation analysis among feature genes was presented *via* Spearman’s correlation test. All statistical p-values calculated were two-sided. Two-sided p < 0.05 was considered to be statistically significant.

## Results

### Evaluation of necroptosis based on snRNA-seq data

**Figure 1:**
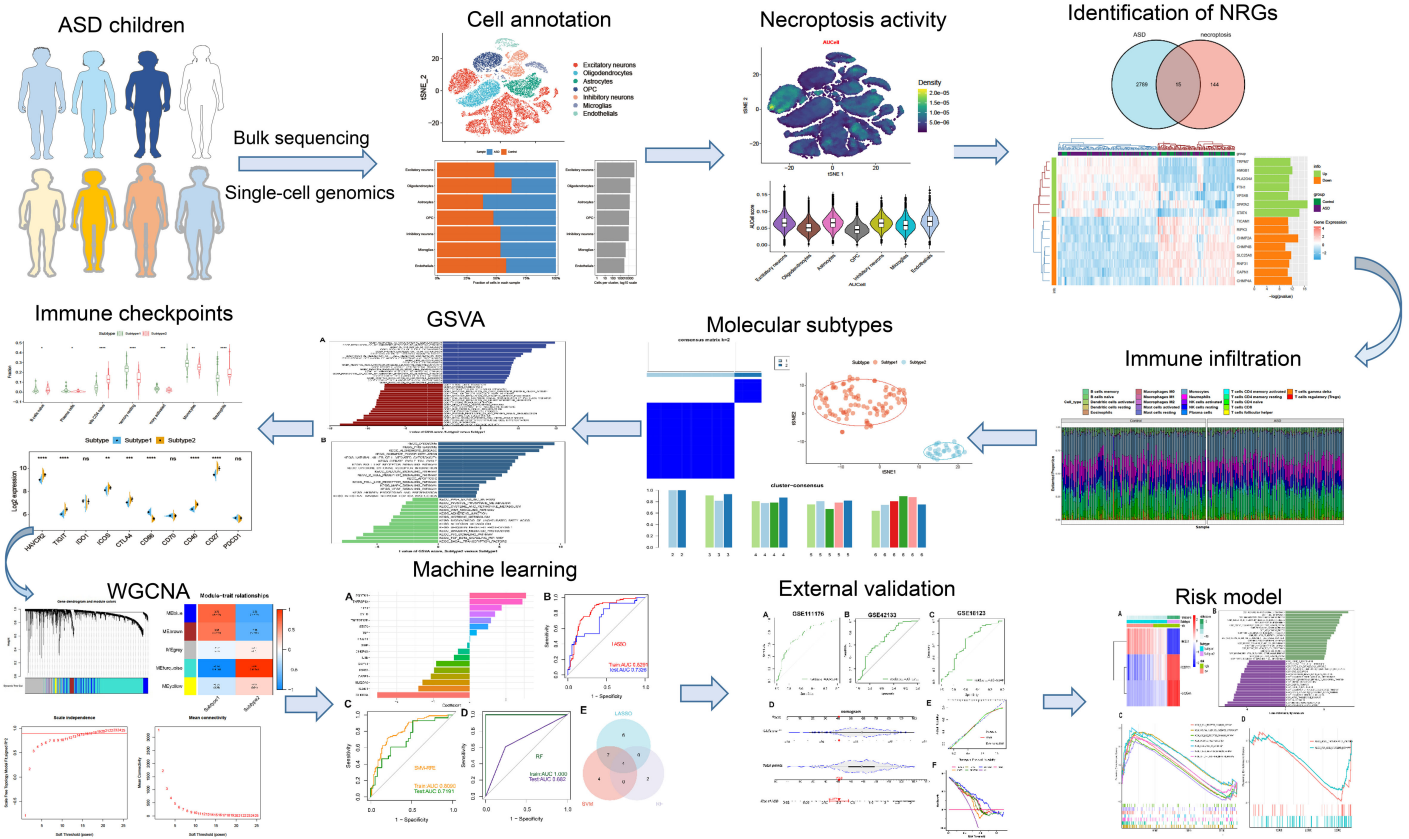
The flow chart of this study. *p < 0.05, **p < 0.01, ***p < 0.001, ****p < 0.0001, and ns no significance.

**Figure 2:**
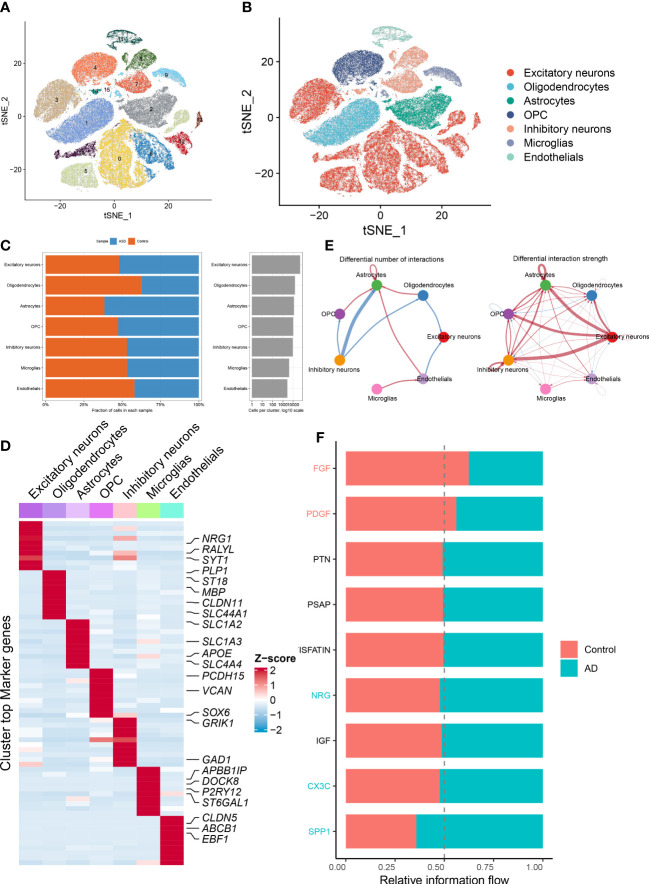
Identification of distinct cell clusters on the basis of snRNA-seq data. **(A, B)** The tSNE clustering of 101420 single cells from 41 cortical samples, exhibited the generation of 16 main clusters **(A)**, including 7 for excitatory neurons, 1 for oligodendrocytes, 1 for astrocytes, 2 for OPC, 3 for inhibitory neurons, 1 for microglias, and 1 for endothelials **(B)**. Each dot corresponds to one cell, and each color corresponds to one cell cluster. **(C)** A stacked bar chart exhibiting the cell type proportions of each group. **(D)** A heatmap showing the top 5 distinctive signature genes in each cluster of cellular annotations. **(E)** Circle plots exhibiting the differences in the number of interactions (left) and strength of interactions (right) in the intercellular communication network between control and ASD groups. The thicker the lines, the stronger the interactions, and the red or blue colors signify increased or decreased signaling pathways in ASD children compared to the control group, respectively. **(F)** A bar graph illustrating the differences in intercellular pathways between the control and ASD groups.

The detailed flow chart of the study process is presented in **Figure 1**. We initially utilized the snRNA-seq expression matrix to explore the extent of necroptosis in ASD patients. Following initial quality control filtering, roughly 36501 distinct genes were extracted from 101420 cells spread over the 41 cortical samples. We performed a PCA and UMAP-based clustering on the informative PCA space (n = 15) after normalizing gene expression, and finally identified 16 distinct cell clusters based on their highly variable genes. Then, these cell clusters were categorized into known cell subtypes as follows: excitatory neurons (48107 cells), oligodendrocytes (15099 cells), astrocytes (11914 cells), OPC (10246 cells), inhibitory neurons (9261 cells), microglias (4055 cells), and endothelials (2738 cells). The tSNE depicted the distribution of cells related to clusters and cell subtypes (**Figures 2A, B**). Cell type proportions of each group are shown in **Figure 2C**, indicating that excitatory neurons, astrocytes, and OPC were enriched in ASD samples. In contrast, oligodendrocytes and endothelials were abundant in control samples. The expression of top 10 signature genes in each cell type are depicted in **Figure 2D**, suggesting that these markers can accurately discriminate distinct cell subtypes. The CellChat analysis was then used to evaluate the differences in intercellular interactions between the control and ASD groups. The interaction strength strengthened from normal to AD. In particular, excitatory neurons with OPC, inhibitory neurons, and astrocytes exhibited greater interaction strengths with other cell types in the ASD group compared to the control group. While less interaction numbers between inhibitory neurons and astrocytes were detected in the ASD group (**Figure 2E**). Comparing the interaction intensities of each pathway led to the generation of specific pathways between the control and ASD groups. NRG, CX3C, and SPP1 signaling pathways are notably active in AD patients, whereas FGF and PDGF signaling pathways are more active in the control group (**Figure 2F**).

**Figure 3:**
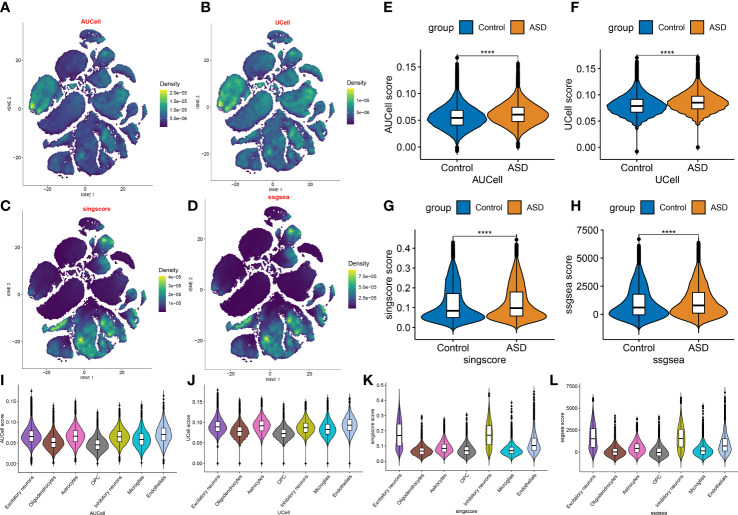
Evaluation of necroptosis activity based on snRNA-seq data. **(A–D)** The AUCell **(A)**, UCell **(B)**, singscore **(C)**, and ssgsea **(D)** algorithms depicting the distribution of necroptosis activity at each cell based on the tSNE plots. **(E–H)** Violin plots exhibiting the differences in necroptosis score between control and ASD groups on the basis of the AUCell **(E)**, UCell **(F)**, singscore **(G)**, and ssgsea **(H)** algorithms. ****p < 0.0001. **(I–L)** Violin plots exhibiting the differences in necroptosis score among various cell subtypes on the basis of the AUCell **(I)**, UCell **(J)**, singscore **(K)**, and ssgsea **(L)** algorithms.

Next, on the basis of the snRNA-seq expression matrix, we implemented a variety of scoring algorithms to quantify the necroptosis score. As illustrated in **Figures 3A–H**, all algorithms, including AUCell, UCell, singscore, and ssgsea, revealed that ASD patients had a considerably greater necroptosis score than the control group, Therefore, we further retrieved and examined snRNA-seq data targeting ASD-infiltrating various cells. Interestingly, we discovered that all four algorithms displayed the prominent necroptosis score in excitatory neurons, inhibitory neurons, and endothelials relative to other cell types (**Figures 3I–L**). These findings indicated that elevated levels of necroptosis may contribute to the progression of ASD.

### Identification of dysregulated necroptosis regulators associated with ASD

**Figure 4:**
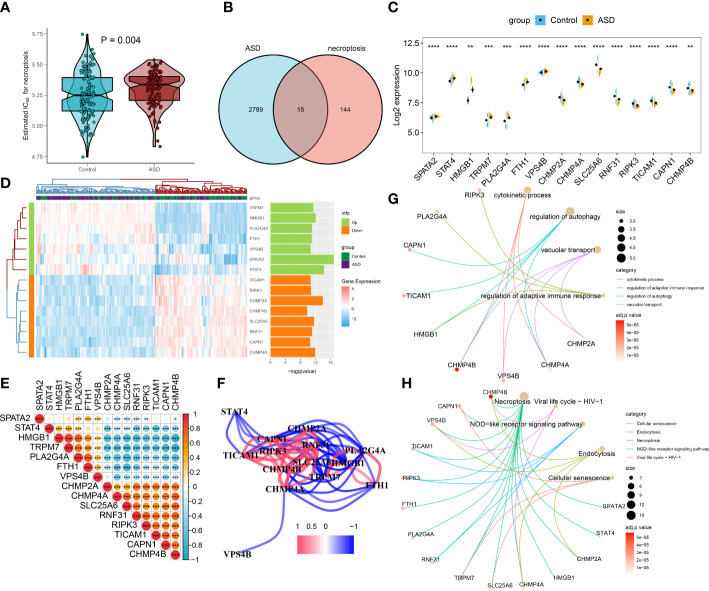
Identification of differential NRGs in ASD children. **(A)** Representative Venn diagram revealing the intersection of DEGs and obtained NRGs. **(B–D)** Representative volcano plot **(B)**, heatmap **(C)**, and split violin plots revealing the differentially expressed NRGs between healthy controls and ASD children. **(E, F)** Representative correlation plot **(E)** and network **(F)** of 15 differentially expressed NRGs. *p < 0.05, ***p < 0.001. **(G, H)** Representative results of GO : BP **(G)** and KEGG **(H)** enrichment analysis. **p < 0.01, and ****p < 0.0001.

We then selected a bulk transcriptome dataset (GSE111176) with 119 ASD and 126 control samples to further validate the necroptosis level in ASD children. Consistently, a significantly higher necroptosis score was also observed in the ASD group (**Figure 4A**). A total of 2804 ASD-related DEGs (1439 up-regulated, 1365 down-regulated) were determined using the DEG approach. Subsequently, we crossed 159 NRGs with 2804 ASD-related DEGs, and finally identified 15 of them as necroptosis-associated DEGs (**Figure 4B**). Among them, the expression levels of SPATA2, STAT4, HMGB1, TRPM7, PLA2G4A, FTH1, and VPS4B genes were markedly higher, while the CHMP2A, CHMP4A, SLC25A6, RNF31, RIPK3, TICAM1, CAPN1 and CHMP4B genes expression levels were notably lower in ASD children than that in non-ASD normal subjects (**Figures 4C, D**). We then correlated these 15 differentially expressed NRGs to assess whether necroptosis exerted toxic effects in ASD children. Interestingly, some NRGs, such as HMGB1 and TRPM7, HMGB1 and PLA2G4A presented highly synergistic actions (coefficient = 0.89 and 0.75). whereas SLC25A6 exhibited significant antagonism effects with HMGB1, TRPM7, PLA2G4A, and FTH1 (coefficient = -0.75, -0.73, -0.81, and -0.75). In addition, the correlation patterns of other NRGs such as RNF31 and CHMP2A, CHMP4A and CHMP4B, RNF31 and CHMP4B, TRPM7 and CHMP4B were also meaningful (**Figures 4E, F**). Furthermore, functional analysis displayed that these NRGS were primarily enriched in cytokinetic processes, regulation of adaptive immune response, regulation of autophagy, and vacuolar transport. The results of KEGG pathway analysis indicated that these NRGS were mainly associated with necroptosis, cellular senescence, endocytosis, HIV-1, and NOD-like receptor signaling pathways (**Figures 4G, H**).

### Altered functions and immunological features in ASD

**Figure 5:**
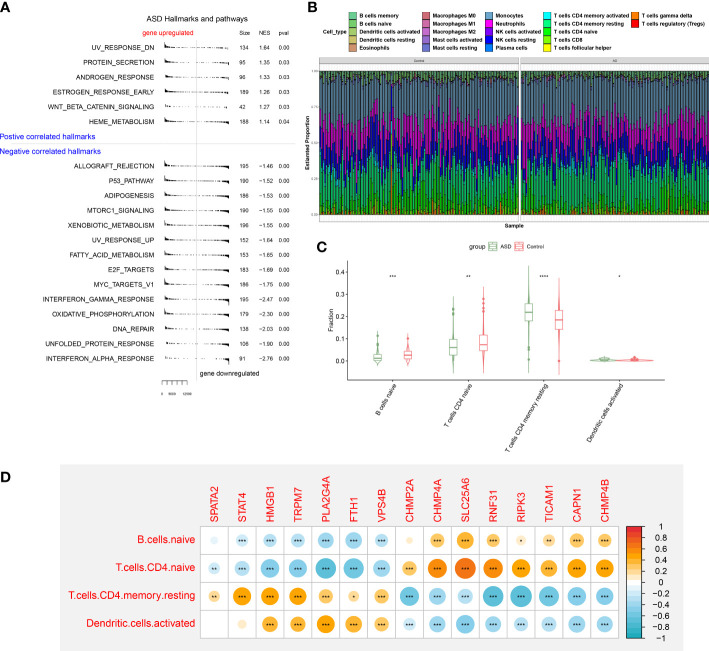
GSEA and immune infiltration analysis between healthy controls and ASD children. **(A)** GSEA showing up- or down-regulated KEGG pathways between healthy individuals and ASD children. A p-value less than 0.05 was considered to be statistically significant. **(B)** Representative stack chart revealing the relative abundances of 22 infiltrated immune cells between healthy individuals and ASD children. **(C)** Representative box plots revealing the infiltration levels of immune cells between healthy individuals and ASD children. **(D)** Representative correlation plot between 15 differentially expressed NRGs and infiltrated immune cells. *p < 0.05, **p < 0.01, ***p < 0.001.

To elucidate whether ASD children exhibited the altered functions and immune patterns, we first performed GSEA and found that oxidative phosphorylation, unfolded protein response, interferon response, and some classic pathways such as p53 and mTORC1 were negatively correlated with ASD (**Figure 5A**). The results of immune infiltration analysis indicated the infiltration levels of naïve B cells, naïve CD4+ T cells, and activated dendritic cells were significantly higher in ASD children (**Figures 5B, C**), suggesting the altered immunological features might be closely related to the onset of ASD. Correlation analysis indicated that most of these 15 NRGs were markedly associated with naïve B cell, naïve CD4+ T cells, activated dendritic cells, and resting memory CD4+ T cells (**Figure 5D**), revealing that the interaction of NRGs with altered immune cells may be the critical pathophysiological mechanism leading to AD progression.

### Identification of necroptosis subtypes in ASD

**Figure 6:**
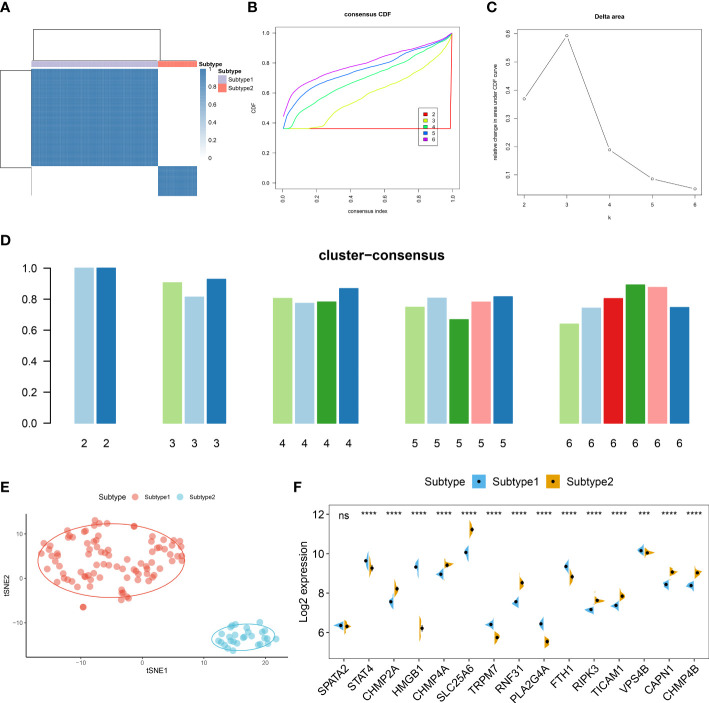
Identification of necroptosis subtypes in ASD. **(A)** Consensus clustering matrices in ASD children (k = 2) based on 15 differentially expressed NRGs. **(B)** Cumulative distribution function (CDF) curves when k = 2–6. **(C)** Relative alterations in the area under CDF curve. **(D)** Consensus clustering score of each subtype. **(E)** t-SNE demonstrating that ASD children are categorized into two distinct necroptosis subtypes. **(F)** Representative split violin plots revealing the expression of 15 differentially expressed NRGs between two subtypes.

To further elucidate the expression patterns of necroptosis in AD, we grouped the 129 ASD children based on the expression profiles of 15 differentially expressed NRGs using a consensus clustering algorithm. The consensus matrix plot showed that the number of subtypes was more stable when k=2 (**Figure 6A**), and the variability of the CDF plot was minimal when the concordance index was 0.2-0.6 (**Figure 6B**). Furthermore, a significant difference in Δ area under the CDF curve was presented when k=2-6 (**Figure 6C**). In addition, when k = 2, each subtype had the highest consistency score (both above 0.9) (**Figure 6D**). Therefore, these ASD samples were grouped into two subtypes, namely subtype1 (n=91) and subtype2 (n=28). tSNE analysis demonstrated a direct and significant distribution difference between these two subtypes (**Figure 6E**). As expected, there was notable heterogeneity in the expression of 14 of 15 NRGs between two subtypes (**Figure 6F**).

### Identification of necroptosis subtypes-associated biological functions and pathways based on GSVA

The differences in enriched functions and signaling pathways between distinct necroptosis expression patterns were assessed by GSVA. The results indicated that mitochondrial-related biological functions, inflammatory responses, and the regulation of immune responses such as T cells extravasation, T cells receptor signaling pathway, antigen processing and presentation, leukocyte mediated immunity, B cells activation and proliferation, and neutrophil migration were prominently upregulated in necroptosis subtype2 (**Figure S1A**). In addition, the results of pathway enrichment analysis revealed that apoptosis, classical pathways, mitochondria, and calcium homeostasis related pathways were elevated in necroptosis suptype2. Otherwise, the significantly enriched pathways also involved immune-related pathways such as the B cells receptor, toll like receptor, natural killer cells, antigen processing and presentation, and the intestinal immune network (**Figure S1B**)

### Identification of necroptosis subtypes-associated immunological features

**Figure 7:**
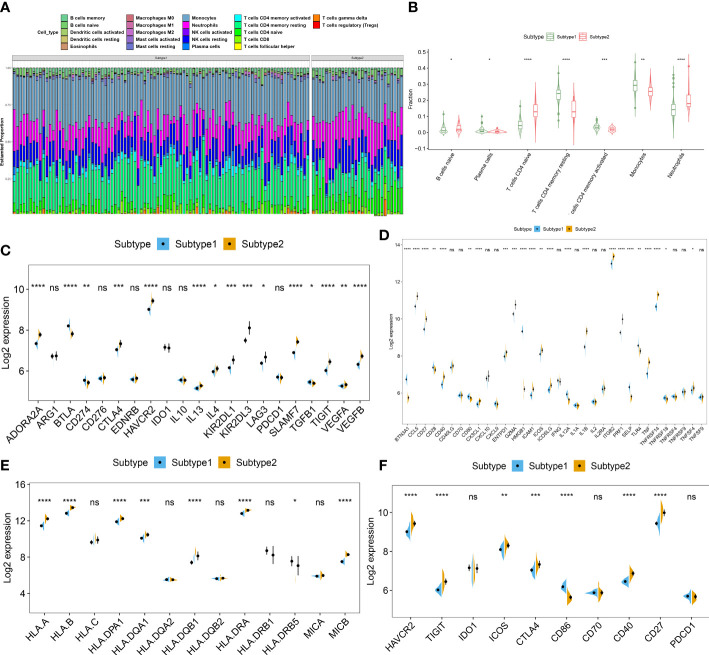
Identification of immunological features between two necroptosis subtypes. **(A)** Representative stack chart revealing the relative abundances of 22 infiltrated immune cells between two necroptosis subtypes. **(B)** Representative box plots revealing the infiltration levels of immune cells between two necroptosis subtypes. **(C–F)** Representative box plots revealing the expression of immunosuppression **(C)**, immune activation **(D)**, MHC-associated genes **(E)**, and immune checkpoints **(F)** between two necroptosis subtypes. *p < 0.05, **p < 0.01, ***p < 0.001, ****p < 0.0001, and ns no significance.

To further clarify the differences in molecular characteristics between two necroptosis subtypes, we then performed immune infiltrating analysis, and the results suggested that Subtype2 exhibited greater proportions of naïve B cells, naïve CD4+ T cells, and neutrophils (**Figures 7A, B**). In addition, we also estimated the difference in classic immune and immune checkpoint-associated genes between two necroptosis subtypes. The results revealed that in comparison to the necroptosis subtype1, most immunosuppression, immune activation, and MHC-associated genes were remarkably upregulated in the necroptosis subtype2 (**Figures 7C–E**). Furthermore, the expression levels of immune checkpoints, such as HAVCR2, TIGIT, ICO2, CTLA4, CD40, and CD27 were notably elevated in the necroptosis subtype2, indicating that the necroptosis subtype2 displayed greater immune responses than necroptosis subtype1. We therefore determined necroptosis subtype2 as an immune subtype and necroptosis subtype1 as a non-immune subtype (**Figure 7F**).

### Co-expression module construction and subtype-specific NGRS identification

**Figure 8:**
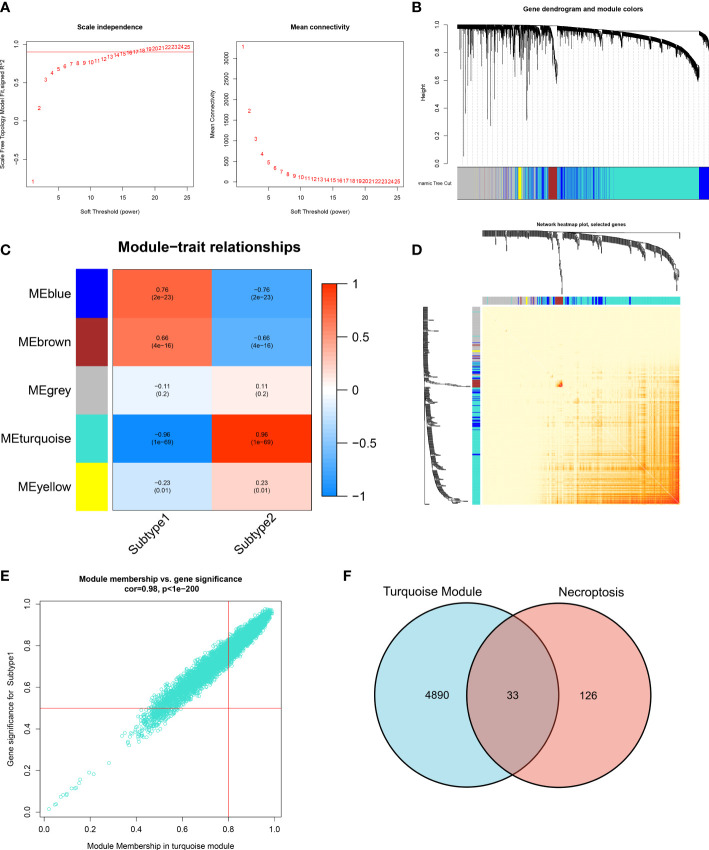
Identification of necroptosis-related NRGs base on WGCNA algorithm. **(A)** Analysis of scale-free fitting index and average connectivity for multiple soft-threshold parameters. **(B)** Representative hierarchical clustering of genes based on the different topological overlaps and module colors. **(C)** Representative module-trait heatmap revealing the correlation of the module eigenvalues with distinct subtypes. **(D)** Representative heatmap of gene networks. **(E)** Representative scatter plot revealing the correlation between turquoise modules and module membership. **(F)** Representative Venn diagram revealing the intersection of turquoise module-related genes and obtained NRGs.

The WGCNA algorithm was employed to construct a co-expression network and identify the module that was most correlated with the subtypes of necroptosis. A network with scale-free topology and connectivity was more efficient when the optimal soft threshold power was set to 15 according to the PickSoftThreshold function (**Figure 8A**). The clustering tree was divided into five different colored modules using a hierarchical clustering approach (**Figure 8B**). Among these modules, the turquoise module (4923 genes) had the highest correlation with subtype1 (R = -0.96) and subtype2 (R = 0.96) (**Figure 8C**). We graphically illustrated the interaction among the identified modules and utilized a heatmap to visualize the TOM containing all genes in the analysis (**Figure 8D**). Meanwhile, there was a notable correlation between turquoise modules and module-related signatures (cor=0.98) (**Figure 8E**). Finally, we identified 33 subtype-specific NGGs by intersecting the turquoise module-related genes with the 159 NRGs obtained from KEGG pathway databases (**Figure 8F**).

### Identification of feature genes *via* LASSO, SVM-RFE, and RF algorithms

**Figure 9:**
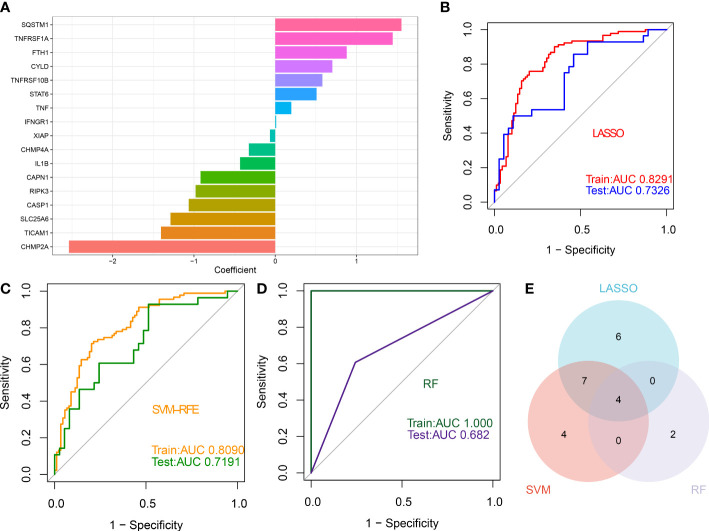
Identification of characteristic NRGs base on machine learning algorithms **(A)** Specific coefficient values for the 17 NRGs identified by the LASSO algorithm. **(B)** ROC curve values for the 17 NRG-based LASSO algorithm in the train and test sets. **(C)** ROC curve values for the 15 NRG-based SVM-RFE algorithm in the train and test sets. **(D)** ROC curve values for the 6 NRG-based RF algorithm in the train and test sets. **(E)** Representative Venn diagram revealing the characteristic NRGs shared by LASSO, RF, and SVM-RFE algorithms.

Three proven machine learning algorithms (LASSO, SVM-RFE, and RF) were applied for identifying key characteristic NRGs. The LASSO algorithm has been cross-validated for five times. Due to the high accuracy of the LASSO classifier base on the optimal lambda (0.0105), we chose the above optimal lambda to construct the LASSO model, and final identified 17 NRGs (CHMP2A, TICAM1, SLC25A6, CASP1, RIPK3, CAPN1, IL1B, CHMP4A, XIAP, IFNGR1, TNF, STAT6, TNFRSF10B, CYLD, FTH1, TNFRSF1A, and SQSTM1) with non-zero coefficients (**Figure 9A**, **Figures S2A, B**). ROC curve analysis suggested that the AUC of the 17-NRG-based LASSO algorithm was 0.8291 in the train set and 0.7326 in the test set (**Figure 9B**). When the optimal number of feature genes for the SVM-RFE algorithm was 15 (SLC25A6, CASP1, CHMP2A, IL1B, SQSTM1, TICAM1, TNFRSF1A, CAPN1, PLA2G4A, CYLD, RIPK3, PLA2G4B, RBCK1, CHMP4A, and TRADD), the classifier had the highest accuracy (**Figure S2C**) and a satisfactory AUC value in both the train set (0.8090) and the test (0.7191) **(** **Figure 9C**). Furthermore, the Boruta feature selection approach confirmed a total of 7 NRGs as important variables (**Figure S2D**). Subsequently, these 7 significant NRGs were fitter into the RF model and achieving an AUC value of 1 in the train set and 0.682 in the test set (**Figure 9D**). A total of 6 NRGs (TRPM7, CHMP4A, TICAM1, CHMP4B, CASP1, and CAPN1) were demonstrated to contribute to the RF model (**Figure S2E**). Following intersection, 4 characteristic NRGs (TICAM1, CASP1, CAPN1, and CHMP4A) shared by LASSO, SVM-RFE, RF algorithms were eventually determined (**Figure 9E**).

### Construction and evaluation of a 4-NRG-based riskScore

**Figure 10:**
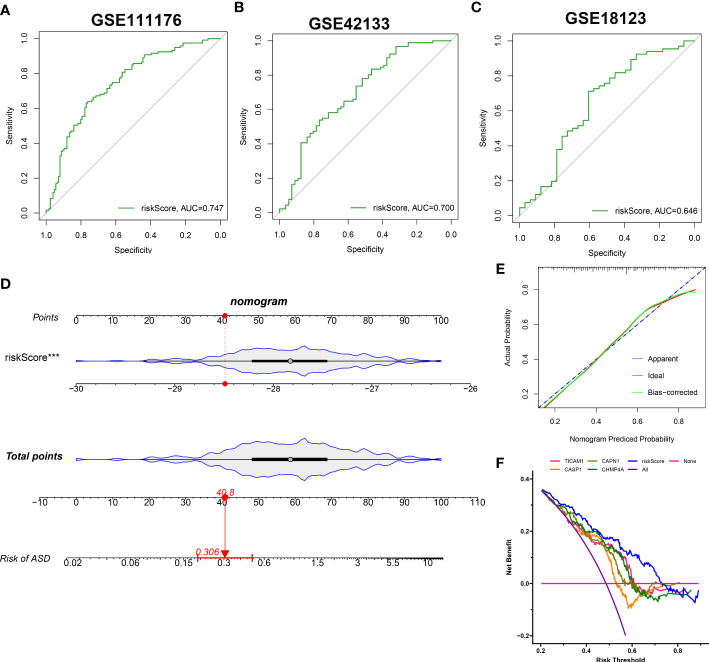
Construction and validation of the performance of 4-NRG diagnostic model. **(A–C)** ROC curves evaluating the diagnostic efficacy of 4-NRG diagnostic model in GSE111176 **(A)**, GSE42133 **(B)**, and GSE18123 **(C)**. **(D)** Representative nomogram based on 4 characteristic NRGs for predicting ASD progression. Each variable corresponds to a score, and the scores of all variables are added together to calculate the total scores. **(E)** Representative calibration curve evaluated the diagnostic accuracy of the nomogram. **(F)** Representative DCA displayed the clinical benefit of the nomogram.

The identified 4 NRGs with corresponding coefficients were utilized to construct a necroptosis-based riskScore as follows: riskScore = (-1.405703 × TICAM1) + (-1.064523 × CASP1) + (-0.919285 × CAPN1) + (-0.324459 × CHMP4A). Afterward, the diagnostic performence of riskScore in predicting the onset of ASD in GSE111176, GSE42133, and GSE18123 was estimated using ROC analysis, and the AUC value of the ROC curve was 0.747, 0.700, and 0.646 in GSE111176, GSE18123, and GSE42133, respectively, proving that the 4-NRG diagnostic model could accurately diagnose ASD to some extent (**Figures 10A–C**). Due to the diagnostic model’s better performance in GSE111176, we next established a nomogram for predicting the risk of ASD in GSE111176 based on the expression landscapes of these 4 NRGs. In the nomogram, the representation of each signature corresponds to a point, and the total points correspond to the different ASD risks, which were obtained by summing the scores of all signatures (**Figure 10D**). The calibration curve demonstrated the accuracy of the nomogram in diagnosing ASD (**Figure 10E**). DCA showed that the clinical application of a nomogram based on the 4-NRG diagnostic model has certain clinical benefits for ASD children (**Figure 10F**).

### Evaluation of the molecular characteristics between distinct risk groups

**Figure 11:**
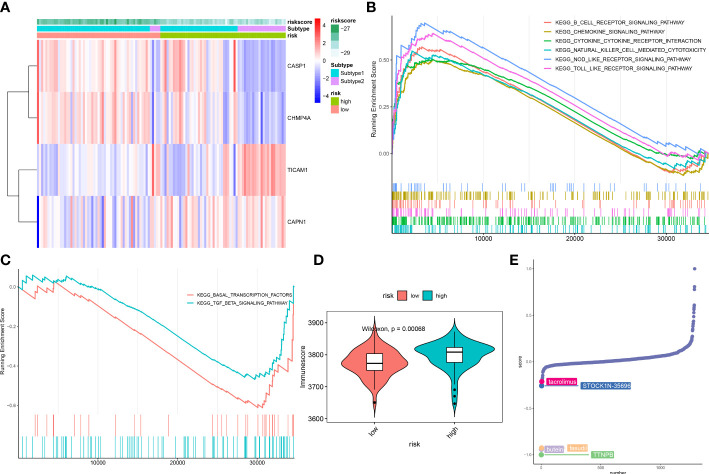
Differences in the molecular characteristics of children at low and high risk of ASD. **(A)** Representative heatmap revealing the expression of model genes in ASD children. **(B, C)** GSEA showing the upregulated **(B)** downregulated **(C)** signaling pathways between low- and high-risk groups. **(D)** Representative violin plot revealing the immunoscore between the low- and high-risk groups. **(E)** CMap analysis revealing the top 5 small molecular compounds targeting high-risk children.

ASD children were classified into high- and low-risk groups based on the median value of constructed riskScore. These 4 NRGs presented distinct expression patterns between the high- and low-risk groups (**Figure 11A**). Next, GSEA was applied to identify the enriched signaling pathways between these two groups. The riskScore based on the 4 NRGs was positively associated with chemokine signaling pathway, cytokine-cytokine receptor interaction, nod like receptor signaling pathway, and immune-related signaling pathways, including B cells receptor, toll like receptor, natural killer cells (**Figure 11B**), whereas it was negatively related to basal transcription factors and TGF-β signaling pathway (**Figure 11C**). Consistently, the high-risk group also presented a higher immune score than that in the low-risk group (**Figure 11D**), suggesting that the high-risk group had more powerful immune responses and might benefit from immune therapy.

To further elucidate the potential drug targets high-necroptosis ASD children, we perform CMap analysis to predict the small-molecule compounds, the result revealed that the top 5 small molecule compounds with potential for individualized treatment for the high-risk group were as follows: tacrolimus, STOCK1N-35696, butein, fasudil, and TTNPB (**Figure 11E**).

External validation of characteristic NRGs by RT-PCR

**Figure 12:**
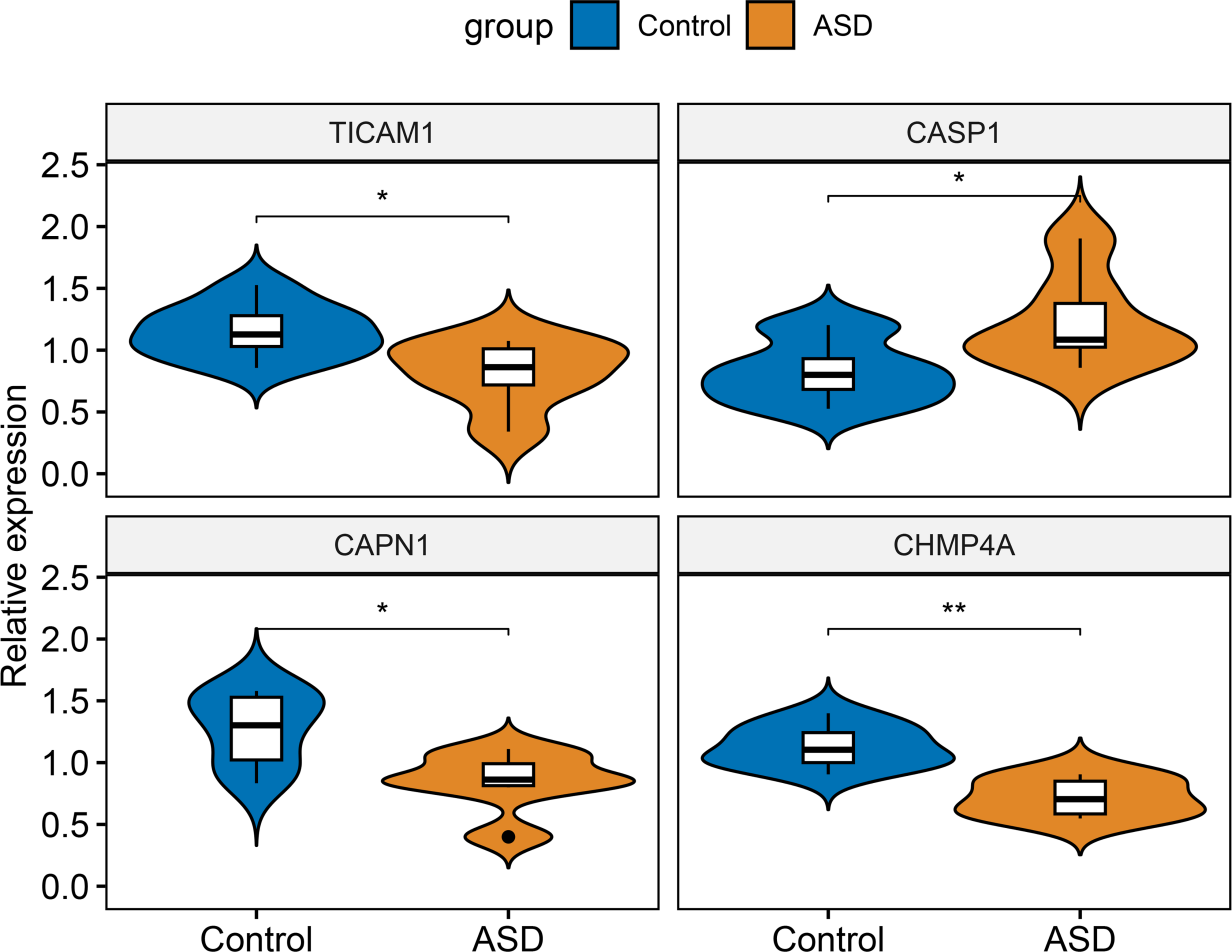
External validation of characteristic NRGs. Representative violin plots revealing the expressional differences in TICAM1, CASP1, CAPN1, and CHMP4A between control and ASD children. *p < 0.05, **p < 0.01.

Finally, RT-PCR was employed to verify the expression landscapes of these four characteristic NRGs in the external cohort. Similarly, the expression levels of CASP1 was notably higher in ASD peripheral blood samples, while TICAM1, CAPN1, and CHMP4A displayed significant downregulation in ASD children (**Figure 12**).

## Discussion

ASD is a neurodevelopmental disorder with a high prevalence in children. Although the Autism Diagnostic Interview-Revised (ADI-R) and the Autism Diagnostic Observation Schedule (ADOS), are currently the most commonly used methods for diagnosing ASD (31), they remain unsatisfactory in terms of diagnostic specificity and accuracy. In addition, due to the lack of specific neurological markers and the heterogeneity of pathogenesis and clinical symptoms in children with ASD, a large number of patients could not achieve effective treatment (32, 33). Thus, further exploration of more accurate diagnostic markers and more representative molecular subtypes associated with ASD is badly needed.

Necrosis is a critical form of programmed cell death characterized by the activation of MLKL/pMLKL through a RIPK1/RIPK3-mediated phosphorylation pathway (13, 34). Recent studies have demonstrated that NRGs play a crucial role in prompting the progression of cardiac and neurological diseases and can serve as a biomarker for prognosis and treatment in patients with multiple diseases (35–38). Furthermore, bioinformatics analysis elucidated the key role of necrosis-based prognostic models in the diagnosis and treatment of patients with various cancers (39–41). However, whether necroptosis is implicated in ASD progression has not been reported so far. Therefore, a large-scale, comprehensive analysis is urgently needed to clarify the role of necroptosis in ASD children. In this study, we first performed snRNA-seq analysis to display the landscape of necroptosis in ASD children, and we found children with ASD exhibited a higher necroptosis score relative to control individuals. Among these distinct cell subtypes, excitatory neurons, inhibitory neurons, and endothelials had the relative higher levels of necroptosis activity. Consistently, bulk transcriptomic data also demonstrated a greater necroptosis score in ASD children. In addition, we determined 15 differentially expressed NRGs between ASD and non-ASD children, most of which exhibited apparent interaction, suggesting a vital role of necroptosis in exacerbating the progression of ASD. Enrichment analysis indicated that these differentially expressed NRGs were mainly involved in immune-related biological functions and signaling pathways. Meanwhile, the abundances of immune cells were significantly different between healthy individuals and ASD children, as evidenced by a higher proportion of naïve B cells, naïve CD4+ T cells, and activated dendritic cells in patients with ASD, which were consistent with the previous studies (42–44). Furthermore, a significant correlation between NRGs and infiltrated immune cells suggested that the potential pathogenic mechanism by which NRGs promoted ASD progression may involve interactions with immune cells. Consensus clustering analysis has been widely applied for identifying distinct subtypes of diseases, especially in tumor patients (45–48). On the basis of 15 differentially expressed NRGs, we classified ASD children into two distinct subtypes using consensus clustering analysis. GSVA enrichment analysis suggested that necroptosis subtype2 was primarily involved in biological functions and pathways including mitochondrial functions, inflammatory responses, and immune responses. Immune infiltration analysis demonstrated a notable difference in infiltrated immune cells between these two necroptosis subtypes. Moreover, higher expression levels of immune checkpoints were also observed in necroptosis subtype2. Combined with these results, it would be reasonable to believe that necroptosis subtype2 may inhibit ASD progression by exhibiting a stronger immune response, and ASD patients with subtype2 may have a better prognosis for immunotherapy.

Subsequently, we identified 33 subtype-specific NRGs based on the WGCNA algorithm. Three machine learning algorithms, including LASSO, SVM-RFE, and RF were performed to determine four characteristic NRGs (TICAM1, CASP1, CAPN1, and CHMP4A). Interestingly, the roles of these four genes in ASD have not been previously reported. TIR domain-containing adaptor molecule-1 (TICAM1), a key regulator of proinflammatory cytokine and interferon (IFN) responses, is a key adapter protein involved in TLR3 and TLR4 signaling pathways. Lack of TICAM1 expression prevents IFN production and is frequently closely associated with human rhinovirus (RV) infection (49). CASP1 is a cell death marker encoding a variety of genes (50). It is reported that the activation of NLRP3-CASP1 axis is closely related to the microglia-mediated neuroinflammation and autophagy dysfunction, eventually leading to the onset of neurological diseases (51). As a member of calpain protein superfamily, CAPN1 activation has been demonstrated to degrade cytoskeleton proteins, vital enzymes, and mitochondrial membrane-related proteins, which may play a primary role in ischemia-induced neuronal injury (52–54). Some research based on microarray analysis has demonstrated that CHMP4A could act as prognostic biomarkers and druggable targets for various diseases, including hepatocellular carcinoma, colorectal cancer, and ovarian carcinoma (55–57). In our current study, we established a risk model composed of 4 characteristics NGRs based on the coefficients of the LASSO analysis. The internal and external validation datasets were utilized to validated the diagnostic performance of these 4 NRGs-based risk model, and the results indicated that the ROC curves, nomograms, calibration curves and DCA based on the four NRGs could predicted the onset of ASD more accurately than individual feature NRG. Combined with the above findings, we considered that necroptosis may be the crucial regulator leading to the poor prognosis and individual heterogeneity in ASD.

To further clarify the correlation of necroptosis with ASD, we grouped ASD children into distinct risk groups based on the median value of riskScore. GSEA revealed that the immune responses-related biological functions and pathways such as B cells receptor, toll like receptor, natural killer cells responses in the high-risk group were stronger than those in the low-risk group. These results revealed that ASD children with a higher necroptosis score might be more likely to benefit from immunotherapy.

However, several limitations cannot be ignored in this study. First, we need to consider more detailed clinical information to verify the clinical effectiveness of the necroptosis score in ASD children. In addition, follow-up experiments *in vivo* or *in vitro* are necessary to explore the underlying roles and mechanisms of these 4 characteristic NRGs in ASD. Furthermore, due to the small sample size, the sensitivity and specificity of the 4-NRG diagnostic model are not satisfactory, which can easily lead to missed diagnosis. Therefore, a larger ASD cohort is urgent to train the diagnostic model and improve the prediction accuracy.

## Conclusion

In summary, we were the first to comprehensively clarify the expression patterns of necroptosis in ASD children and to disclose a novel molecular classification associated with necroptosis. In addition, machine learning-based determination of four characteristic NRGs (TICAM1, CASP1, CAPN1, and CHMP4A) could accurately predict ASD onset. Moreover, we constructed a riskScore model on the basis of characteristic NRGs and explored potential small-molecule compounds targeting ASD children at different risks. Our study would provide novel insights into the early diagnosis and individualized treatment of children with ASD.

## Data availability statement

The original contributions presented in the study are included in the article/**Supplementary Material**. Further inquiries can be directed to the corresponding authors.

## Ethics statement

The studies involving human participants were reviewed and approved by the Ethics Committee of Fujian Children’s Hospital. Written informed consent to participate in this study was provided by the participants’ legal guardian/next of kin.

## Author contributions

The study was designed by LL and YL. Statistical analyses were conducted by HJ, HD, and ZZ. Original manuscript was written by LL and QF. The funding was provided and the study was supervised by YL. The data was collected by QF, ZZ, and HD. The figures were prepared by LL and QF. The manuscript was revised by YL. All authors contributed to the article and approved the submitted version.

## References

[1] Lord C Elsabbagh M Baird G Veenstra-Vanderweele J. Autism spectrum disorder. Lancet (2018) 392(10146):508–20. doi: 10.1016/s0140-6736(18)31129-2 PMC7398158 30078460

[2] Hu T Dong Y He C Zhao M He Q. The gut microbiota and oxidative stress in autism spectrum disorders (ASD). Oxid Med Cell Longev (2020) 2020:8396708. doi: 10.1155/2020/8396708 33062148 PMC7547345

[3] Rubenstein JL Merzenich MM. Model of autism: increased ratio of excitation/inhibition in key neural systems. Genes Brain Behav (2003) 2(5):255–67. doi: 10.1034/j.1601-183x.2003.00037.x PMC6748642 14606691

[4] Wang W Tang J Zhong M Chen J Li T Dai Y. HIF-1 α may play a role in late pregnancy hypoxia-induced autism-like behaviors in offspring rats. Behav Brain Res (2021) 411:113373. doi: 10.1016/j.bbr.2021.113373 34048873

[5] Hadders-Algra M. Emerging signs of autism spectrum disorder in infancy: putative neural substrate. Dev Med Child Neurol (2022) 64(11):1344–50. doi: 10.1111/dmcn.15333 PMC9796067 35801808

[6] Mehta R Kuhad A Bhandari R. Nitric oxide pathway as a plausible therapeutic target in autism spectrum disorders. Expert Opin Ther Targets (2022) 26(7):1–21. doi: 10.1080/14728222.2022.2100252 35811505

[7] Thawley AJ Veneziani LP Rabelo-da-Ponte FD Riederer I Mendes-da-Cruz DA Bambini-Junior V. Aberrant IL-17 levels in rodent models of autism spectrum disorder: a systematic review. Front Immunol (2022) 13:874064. doi: 10.3389/fimmu.2022.874064 35757754 PMC9226456

[8] Karlowitz R van Wijk SJL. Surviving death: emerging concepts of RIPK3 and MLKL ubiquitination in the regulation of necroptosis. FEBS J (2021) 290(1):37–54. doi: 10.1111/febs.16255 34710282

[9] Horne CR Samson AL Murphy JM. The web of death: the expanding complexity of necroptotic signaling. Trends Cell Biol (2022) 33(2):162–74. doi: 10.1016/j.tcb.2022.05.008 35750616

[10] Salem D Subang R Pernet E Divangahi M Pineau C Cayrol R. Necroptotic cell binding of β(2) -glycoprotein I provides a potential autoantigenic stimulus in systemic lupus erythematosus. Immunol Cell Biol (2019) 97(9):799–814. doi: 10.1111/imcb.12279 31187539

[11] Zhao J Jiang P Guo S Schrodi SJ He D. Apoptosis, autophagy, NETosis, necroptosis, and pyroptosis mediated programmed cell death as targets for innovative therapy in rheumatoid arthritis. Front Immunol (2021) 12:809806. doi: 10.3389/fimmu.2021.809806 35003139 PMC8739882

[12] Wu Z Deng J Zhou H Tan W Lin L Yang J. Programmed cell death in sepsis associated acute kidney injury. Front Med (Lausanne) (2022) 9:883028. doi: 10.3389/fmed.2022.883028 35655858 PMC9152147

[13] Zang X Song J Li Y Han Y. Targeting necroptosis as an alternative strategy in tumor treatment: from drugs to nanoparticles. J Control Release (2022) 349:213–26. doi: 10.1016/j.jconrel.2022.06.060 35793737

[14] Tao Y Murakami Y Vavvas DG Sonoda KH. Necroptosis and neuroinflammation in retinal degeneration. Front Neurosci (2022) 16:911430. doi: 10.3389/fnins.2022.911430 35844208 PMC9277228

[15] Thadathil N Nicklas EH Mohammed S Lewis TL Jr. Richardson A Deepa SS. Necroptosis increases with age in the brain and contributes to age-related neuroinflammation. Geroscience (2021) 43(5):2345–61. doi: 10.1007/s11357-021-00448-5 PMC8599532 34515928

[16] Jun-Long H Yi L Bao-Lian Z Jia-Si L Ning Z Zhou-Heng Y. Necroptosis signaling pathways in stroke: from mechanisms to therapies. Curr Neuropharmacol. (2018) 16(9):1327–39. doi: 10.2174/1570159x16666180416152243 PMC6251040 29663889

[17] Davis S Meltzer PS. GEOquery: a bridge between the gene expression omnibus (GEO) and BioConductor. Bioinformatics (2007) 23(14):1846–7. doi: 10.1093/bioinformatics/btm254 17496320

[18] Velmeshev D Schirmer L Jung D Haeussler M Perez Y Mayer S. Single-cell genomics identifies cell type-specific molecular changes in autism. Science (2019) 364(6441):685–9. doi: 10.1126/science.aav8130 PMC7678724 31097668

[19] Jin S Guerrero-Juarez CF Zhang L Chang I Ramos R Kuan CH. Inference and analysis of cell-cell communication using CellChat. Nat Commun (2021) 12(1):1088. doi: 10.1038/s41467-021-21246-9 33597522 PMC7889871

[20] Yu G Wang LG Han Y He QY. clusterProfiler: an r package for comparing biological themes among gene clusters. Omics (2012) 16(5):284–7. doi: 10.1089/omi.2011.0118 PMC3339379 22455463

[21] Hänzelmann S Castelo R Guinney J. GSVA: gene set variation analysis for microarray and RNA-seq data. BMC Bioinf (2013) 14:7. doi: 10.1186/1471-2105-14-7 PMC3618321 23323831

[22] Newman AM Steen CB Liu CL Gentles AJ Chaudhuri AA Scherer F. Determining cell type abundance and expression from bulk tissues with digital cytometry. Nat Biotechnol (2019) 37(7):773–82. doi: 10.1038/s41587-019-0114-2 PMC6610714 31061481

[23] Wilkerson MD Hayes DN. ConsensusClusterPlus: a class discovery tool with confidence assessments and item tracking. Bioinformatics (2010) 26(12):1572–3. doi: 10.1093/bioinformatics/btq170 PMC2881355 20427518

[24] Langfelder P Horvath S. WGCNA: an r package for weighted correlation network analysis. BMC Bioinf (2008) 9:559. doi: 10.1186/1471-2105-9-559 PMC2631488 19114008

[25] Bøvelstad HM Nygård S Størvold HL Aldrin M Borgan Ø Frigessi A. Predicting survival from microarray data–a comparative study. Bioinformatics (2007) 23(16):2080–7. doi: 10.1093/bioinformatics/btm305 17553857

[26] Hu X Martinez-Ledesma E Zheng S Kim H Barthel F Jiang T. Multigene signature for predicting prognosis of patients with 1p19q co-deletion diffuse glioma. Neuro Oncol (2017) 19(6):786–95. doi: 10.1093/neuonc/now285 PMC5464432 28340142

[27] Engebretsen S Bohlin J. Statistical predictions with glmnet. Clin Epigenet (2019) 11(1):123. doi: 10.1186/s13148-019-0730-1 PMC6708235 31443682

[28] Huang ML Hung YH Lee WM Li RK Jiang BR. SVM-RFE based feature selection and taguchi parameters optimization for multiclass SVM classifier. ScientificWorldJournal (2014) 2014:795624. doi: 10.1155/2014/795624 25295306 PMC4175386

[29] Xu H Zhao B Zhong W Teng P Qiao H. Identification of miRNA signature associated with erectile dysfunction in type 2 diabetes mellitus by support vector machine-recursive feature elimination. Front Genet (2021) 12:762136. doi: 10.3389/fgene.2021.762136 34707644 PMC8542849

[30] Liaw A Wiener M. Classification and regression by randomForest. R News (2002) 2(3):18–22.

[31] Hong SJ Vos de Wael R Bethlehem RAI Lariviere S Paquola C Valk SL. Atypical functional connectome hierarchy in autism. Nat Commun (2019) 10(1):1022. doi: 10.1038/s41467-019-08944-1 30833582 PMC6399265

[32] Anderson GM Scahill L McCracken JT McDougle CJ Aman MG Tierney E. Effects of short- and long-term risperidone treatment on prolactin levels in children with autism. Biol Psychiatry (2007) 61(4):545–50. doi: 10.1016/j.biopsych.2006.02.032 16730335

[33] Ospina MB Krebs Seida J Clark B Karkhaneh M Hartling L Tjosvold L. Behavioural and developmental interventions for autism spectrum disorder: a clinical systematic review. PloS One (2008) 3(11):e3755. doi: 10.1371/journal.pone.0003755 19015734 PMC2582449

[34] Pasparakis M Vandenabeele P. Necroptosis and its role in inflammation. Nature (2015) 517(7534):311–20. doi: 10.1038/nature14191 25592536

[35] DeRoo E Zhou T Liu B. The role of RIPK1 and RIPK3 in cardiovascular disease. Int J Mol Sci (2020) 21(21):8174. doi: 10.3390/ijms21218174 33142926 PMC7663726

[36] Cui J Zhao S Li Y Zhang D Wang B Xie J. Regulated cell death: discovery, features and implications for neurodegenerative diseases. Cell Commun Signal (2021) 19(1):120. doi: 10.1186/s12964-021-00799-8 34922574 PMC8684172

[37] Zhou Y Liao J Mei Z Liu X Ge J. Insight into crosstalk between ferroptosis and necroptosis: novel therapeutics in ischemic stroke. Oxid Med Cell Longev (2021) 2021:9991001. doi: 10.1155/2021/9991001 34257829 PMC8257382

[38] Li S Qu L Wang X Kong L. Novel insights into RIPK1 as a promising target for future alzheimer’s disease treatment. Pharmacol Ther (2022) 231:107979. doi: 10.1016/j.pharmthera.2021.107979 34480965

[39] Zhao C Xiong K Adam A Ji Z Li X. Necroptosis identifies novel molecular phenotypes and influences tumor immune microenvironment of lung adenocarcinoma. Front Immunol (2022) 13:934494. doi: 10.3389/fimmu.2022.934494 35911707 PMC9331758

[40] Xie J Chen L Tang Q Wei W Cao Y Wu C. A necroptosis-related prognostic model of uveal melanoma was constructed by single-cell sequencing analysis and weighted Co-expression network analysis based on public databases. Front Immunol (2022) 13:847624. doi: 10.3389/fimmu.2022.847624 35242144 PMC8886618

[41] Xie J Tian W Tang Y Zou Y Zheng S Wu L. Establishment of a cell necroptosis index to predict prognosis and drug sensitivity for patients with triple-negative breast cancer. Front Mol Biosci (2022) 9:834593. doi: 10.3389/fmolb.2022.834593 35601830 PMC9117653

[42] Basheer S Venkataswamy MM Christopher R Van Amelsvoort T Srinath S Girimaji SC. Immune aberrations in children with autism spectrum disorder: a case-control study from a tertiary care neuropsychiatric hospital in India. Psychoneuroendocrinology (2018) 94:162–7. doi: 10.1016/j.psyneuen.2018.05.002 29804052

[43] Al-Harbi NO Nadeem A Ahmad SF Al-Ayadhi LY Al-Harbi MM As Sobeai HM. Elevated expression of toll-like receptor 4 is associated with NADPH oxidase-induced oxidative stress in b cells of children with autism. Int Immunopharmacol. (2020) 84:106555. doi: 10.1016/j.intimp.2020.106555 32388012

[44] De Giacomo A Gargano CD Simone M Petruzzelli MG Pedaci C Giambersio D. B and T immunoregulation: a new insight of b regulatory lymphocytes in autism spectrum disorder. Front Neurosci (2021) 15:732611. doi: 10.3389/fnins.2021.732611 34776843 PMC8581677

[45] Qiu X Hua X Li Q Zhou Q Chen J. m(6)A regulator-mediated methylation modification patterns and characteristics of immunity in blood leukocytes of COVID-19 patients. Front Immunol (2021) 12:774776. doi: 10.3389/fimmu.2021.774776 34917088 PMC8669770

[46] Shao W Yang Z Fu Y Zheng L Liu F Chai L. The pyroptosis-related signature predicts prognosis and indicates immune microenvironment infiltration in gastric cancer. Front Cell Dev Biol (2021) 9:676485. doi: 10.3389/fcell.2021.676485 34179006 PMC8226259

[47] Zhang W Yao S Huang H Zhou H Zhou H Wei Q. Molecular subtypes based on ferroptosis-related genes and tumor microenvironment infiltration characterization in lung adenocarcinoma. Oncoimmunology (2021) 10(1):1959977. doi: 10.1080/2162402x.2021.1959977 34527427 PMC8437492

[48] Zheng Y Yue X Fang C Jia Z Chen Y Xie H. A novel defined endoplasmic reticulum stress-related lncRNA signature for prognosis prediction and immune therapy in glioma. Front Oncol (2022) 12:930923. doi: 10.3389/fonc.2022.930923 35847925 PMC9282894

[49] Tao S Zhu L Lee P Lee WM Knox K Chen J. Negative control of TLR3 signaling by TICAM1 down-regulation. Am J Respir Cell Mol Biol (2012) 46(5):660–7. doi: 10.1165/rcmb.2011-0340OC PMC3359907 22205631

[50] Reinke S Linge M Diebner HH Luksch H Glage S Gocht A. Non-canonical caspase-1 signaling drives RIP2-dependent and TNF-α-Mediated inflammation *In Vivo* . Cell Rep (2020) 30(8):2501–2511.e2505. doi: 10.1016/j.celrep.2020.01.090 32101731

[51] Wang D Zhang J Jiang W Cao Z Zhao F Cai T. The role of NLRP3-CASP1 in inflammasome-mediated neuroinflammation and autophagy dysfunction in manganese-induced, hippocampal-dependent impairment of learning and memory ability. Autophagy (2017) 13(5):914–27. doi: 10.1080/15548627.2017.1293766 PMC5446056 28318352

[52] Yamakawa H Banno Y Nakashima S Yoshimura S Sawada M Nishimura Y. Crucial role of calpain in hypoxic PC12 cell death: calpain, but not caspases, mediates degradation of cytoskeletal proteins and protein kinase c-alpha and -delta. Neurol Res (2001) 23(5):522–30. doi: 10.1179/016164101101198776 11474809

[53] Wu HY Yuen EY Lu YF Matsushita M Matsui H Yan Z. Regulation of n-methyl-D-aspartate receptors by calpain in cortical neurons. J Biol Chem (2005) 280(22):21588–93. doi: 10.1074/jbc.M501603200 15790561

[54] Liu Y Che X Zhang H Fu X Yao Y Luo J. CAPN1 (Calpain1)-mediated impairment of autophagic flux contributes to cerebral ischemia-induced neuronal damage. Stroke (2021) 52(5):1809–21. doi: 10.1161/strokeaha.120.032749 33874744

[55] Barlin JN Jelinic P Olvera N Bogomolniy F Bisogna M Dao F. Validated gene targets associated with curatively treated advanced serous ovarian carcinoma. Gynecol Oncol (2013) 128(3):512–7. doi: 10.1016/j.ygyno.2012.11.018 23168173

[56] Li H Li T Zhang X. Identification of a pyroptosis-related prognostic signature combined with experiments in hepatocellular carcinoma. Front Mol Biosci (2022) 9:822503. doi: 10.3389/fmolb.2022.822503 35309514 PMC8931679

[57] Ma L Yu H Zhu Y Xu K Zhao A Ding L. Isolation and proteomic profiling of urinary exosomes from patients with colorectal cancer. Proteome Sci (2023) 21(1):3. doi: 10.1186/s12953-023-00203-y 36759883 PMC9909931

