# Supplementary material for: Combination of machine learning-based bulk and single-cell genomics reveals necroptosis-related molecular subtypes and immunological features in autism spectrum disorder
Source: Front Immunol. 2023 Apr 24;14:1139420. doi: 10.3389/fimmu.2023.1139420 (PMC10165081; doi:10.3389/fimmu.2023.1139420)
Supplement: Supplementary file 1 [file DataSheet_1.docx]

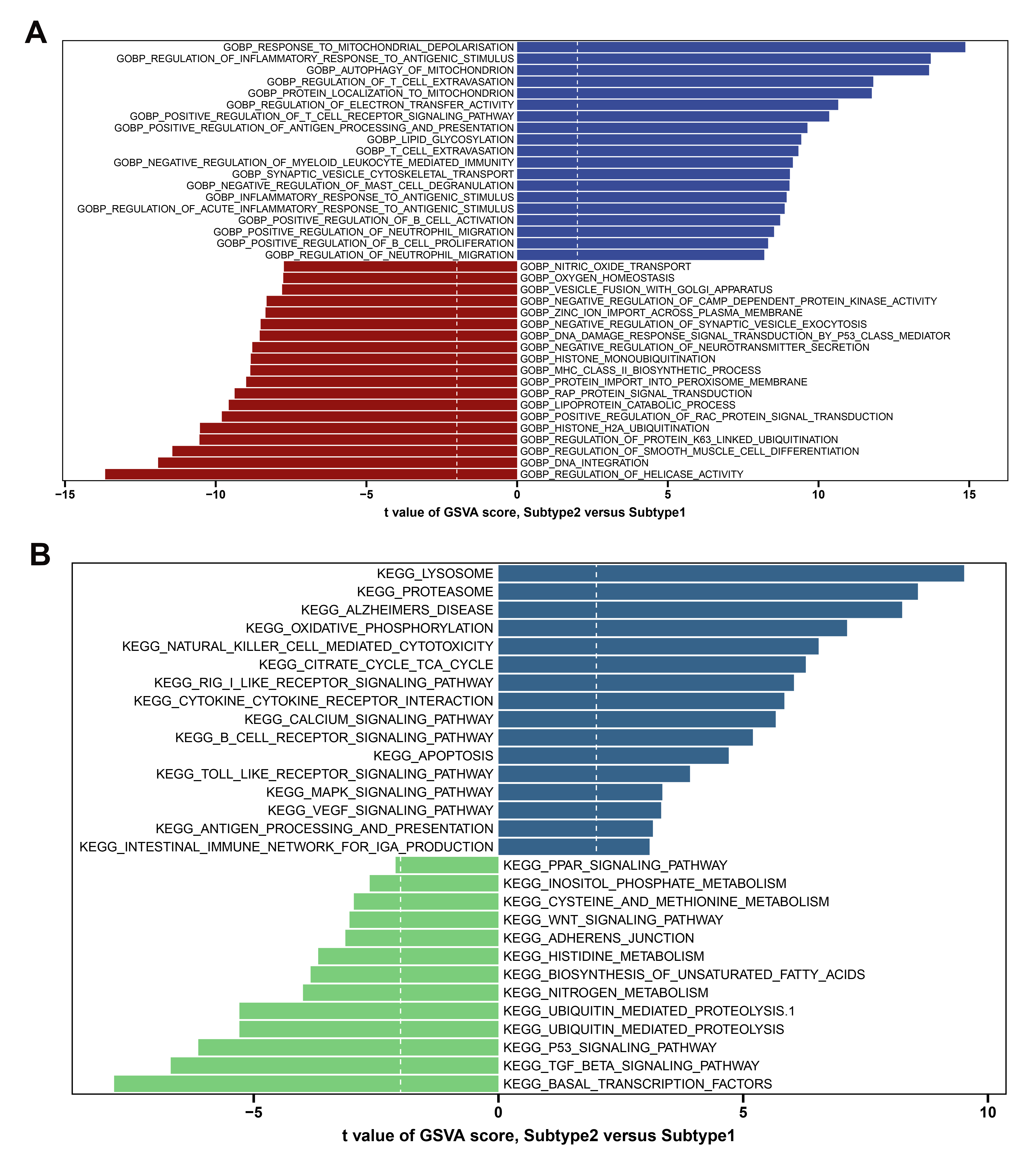


**Figure S1 Enrichment analysis between two necroptosis subtypes.** (A,B) GSVA revealing enriched biological processes (A) and signaling pathways (B)**.**


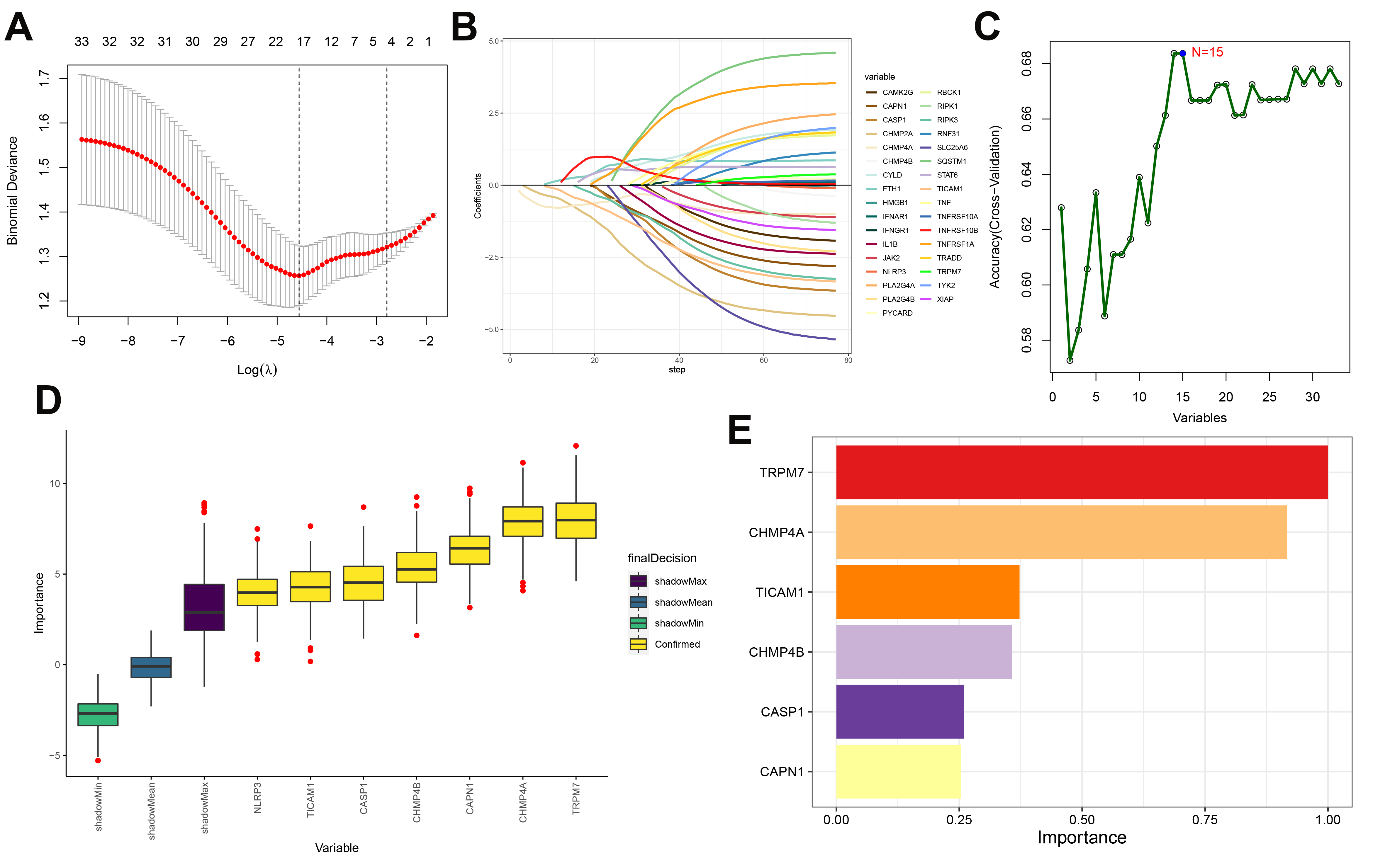


**Figure S2 Identification of characteristic NRGs base on machine learning algorithms.** (A) Selection of the optimal lambda value in the LASSO algorithm on the basis of five-fold cross-validation. (B) The LASSO coefficient profiling of the 9 NRGs with nonzero coefficients screened by the optimal lambda. (C) SVM-RFE algorithm for feature selection based on the cross-validated accuracy. (D) A total of 7 NRGs were identified as important variables using the Boruta algorithm. (E) RF algorithm-based ranking of the relative importance of genes.
